# Diffusing capacity of the lung for carbon monoxide, transfer coefficient of the lung for carbon monoxide and forced vital capacity/diffusing capacity of the lung for carbon monoxide in suspected systemic sclerosis-associated pulmonary hypertension: insights from the ASPIRE registry

**DOI:** 10.1183/23120541.00798-2025

**Published:** 2026-03-23

**Authors:** Howard Smith, A.A. Roger Thompson, Mohammed Akil, Samer Alabed, Catherine Billings, Athanasios Charalampopoulos, Krit Dwivedi, Charlie A. Elliot, Abdul Hameed, Ashraful Haque, Neil Hamilton, Catherine Hill, Judith Hurdman, Rachael Kilding, Kar-Ping Kuet, Smitha Rajaram, Alexander M.K. Rothman, Ian Smith, Andrew J. Swift, David G. Kiely, Robin Condliffe

**Affiliations:** 1 Sheffield Pulmonary Vascular Disease Unit, Royal Hallamshire Hospital, Sheffield, UK; 2 Division of Clinical Medicine, School of Medicine and Population Health, University of Sheffield, Sheffield, UK; 3 Department of Rheumatology, Royal Hallamshire Hospital, Sheffield, UK; 4 Department of Radiology, Royal Hallamshire Hospital, Sheffield, UK; 5 National Institute for Health and Care Research Sheffield Biomedical Research Centre, Sheffield, UK; 6 Insigneo Institute, University of Sheffield, Sheffield, UK

## Abstract

**Objectives:**

There are limited data comparing parameters reflecting gas transfer used to assess the likelihood of pulmonary hypertension (PH) in patients with systemic sclerosis (SSc) and regarding the impact of transitioning to Global Lung Initiative (GLI)-predicted values.

**Methods:**

632 patients with suspected SSc-associated PH were identified from the ASPIRE registry. Spirometry and computed tomography reports were reviewed to identify significant lung disease. Receiver operating characteristic curve analysis and correlations of the three markers of gas transfer with pulmonary arterial pressure were performed.

**Results:**

Correlations of GLI-derived values with mean pulmonary arterial pressure were diffusing capacity of the lung for carbon monoxide (*D*_LCO_)% r= −0.45, transfer coefficient of the lung for carbon monoxide (*K*_CO_)% r= −0.42 and forced vital capacity (FVC)%/*D*_LCO_% r=0.37. Correlations in patients without lung disease were *D*_LCO_% r= −0.51, *K*_CO_% r= −0.44, FVC%/*D*_LCO_% r=0.38, compared with patients with lung disease: *D*_LCO_% r= −0.41, *K*_CO_% r= −0.39, FVC%/*D*_LCO_% r=0.39. Area under the curve for the presence of PH in the overall study cohort was significantly superior for *D*_LCO_% at 0.84 (optimal threshold 53%), compared with *K*_CO_% 0.74 (60%) and FVC%/*D*_LCO_% was 0.74 (1.91), p<0.001 for both. Compared with European Coal and Steel Community-derived data, GLI-derived % predicted lung volumes were lower, *D*_LCO_% and *K*_CO_% were higher and consequently FVC%/*D*_LCO_% lower (p<0.001, all).

**Conclusion:**

*D*_LCO_ performed as least as strongly as *K*_CO_ or FVC%/ *D*_LCO_% in terms of correlations with mean pulmonary arterial pressure and diagnostic utility, regardless of the presence or absence of lung disease. Transitioning to GLI equations led to lower predicted spirometric volumes and higher *D*_LCO_%. This should be considered when interpreting changes in values over time and when using screening algorithms.

## Shareable abstract

***D*_LCO_ is non-inferior to other measures of gas transfer in predicting the presence of PH in patients with systemic sclerosis. Changing from ECSC to GLI prediction equations results in lower FVC%/*D*_LCO_%: this should be considered when using prediction tools.** https://bit.ly/4nKK4A3

## Introduction

Pulmonary hypertension (PH) may develop for a number of reasons, including the presence of a pulmonary arterial vasculopathy (pulmonary arterial hypertension; PAH) or in association with chronic lung disease (CLD) [1, 2]. Although PAH is a rare condition in the general population it develops in 6.4–9% of patients with systemic sclerosis (SSc) [3, 4]. Patients with SSc may also develop PH-CLD given that ≈40% of patients have clinically overt interstitial lung disease (ILD) [5]. A number of screening algorithms for the early identification of SSc-associated PH have been developed including the DETECT and Australian Scleroderma Interest Group approaches, both of which include a measure of gas transfer [6, 7].

The diffusing capacity for carbon monoxide (*D*_LCO_) is assessed most commonly *via* the single breath method [8]. During this, carbon monoxide is removed exponentially from alveolar gas at a rate constant (*K*_CO_), while alveolar volume (*V*_A_) is calculated from dilution of an inert gas, most often helium. The product of *K*_CO_ and *V*_A_ is termed *D*_LCO_. The ratio of forced vital capacity (FVC) % predicted (FVC%) to *D*_LCO_ % predicted (*D*_LCO_%), FVC%/*D*_LCO_%, has been proposed as a superior measure of gas transfer in patients with SSc, especially those with coexisting ILD [9]. *D*_LCO_% tends to be lower in patients with SSc-PAH than in patients with idiopathic PAH, while a recent study demonstrated an accelerated fall in *D*_LCO_% and *K*_CO_% and accelerated rise in FVC%/*D*_LCO_% in the ≈6 years prior to diagnosis of PH [10, 11]. There are, however, limited published data directly comparing these three markers of gas transfer in SSc patients or examining the effect of changing clinical practice from the use of European Coal and Steel Community (ECSC) to Global Lung Function Initiative (GLI) predictive equations in patients with SSc [12, 13, 14].

We have therefore interrogated a large database of patients assessed at a specialist referral centre (Assessing the Spectrum of Pulmonary hypertension Identified at a REferral centre; ASPIRE) to test the hypothesis that *K*_CO_% or FVC%/*D*_LCO_% are superior, both in terms of diagnostic utility and correlation with pulmonary arterial pressure, to *D*_LCO_% in patients with suspected SSc-associated pre-capillary PH. We also investigated the effect of transitioning from ECSC to GLI reference equations.

## Methods

The ASPIRE registry, which consists of consecutive patients reviewed at a UK PH referral centre, was interrogated to identify consecutive patients with SSc who had been investigated during 2000–2020 [15, 16]. Demographic, haemodynamic and lung function data were collected from clinical datasets.

Haemodynamic diagnostic criteria described in the 2022 European Society of Cardiology/European Respiratory Society (ESC/ERS) PH guidelines were used [17]. In brief, PH was defined by a mean pulmonary arterial pressure (mPAP) >20 mmHg. Pre-capillary PH (PAH or PH-CLD) was defined by a pulmonary arterial wedge pressure (PAWP) ≤15 mmHg and a pulmonary vascular resistance (PVR) >2 WU, PH due to left heart disease (PH-LHD) by a PAWP >15 mmHg and Unclassified-PH by a PAWP ≤15 mmHg and PVR ≤2 WU. The date of diagnosis was the date of the first right heart catheterisation, which demonstrated the presence or absence of PH. Ethical approval was gained (REC 22/EE/0011).

Pulmonary function testing was performed according to contemporary ERS guidelines with *D*_LCO_ measured using the single breath technique [18–23]. Prior to 2020, in our service ECSC reference equations were used to produce reference values for individual patients [13, 21]. During 2020, reference values began to be calculated using GLI data [14, 24]. For the purpose of this study, reference values for spirometry and measures of gas diffusion capacity for carbon monoxide (uncorrected for haemoglobin levels) were derived using both ECSC and GLI Global equations [25]. Patients who had incomplete data to allow calculation of both ECSC and GLI reference values or who had undergone lung function testing >90 days before or after the date of right heart catheterisation (RHC) were excluded.

Computed tomography (CT) clinical reports nearest to the time of diagnosis were retrieved and the extent of any parenchymal lung disease was recorded as being minor, mild, moderate or severe. If no reports were available, then descriptions from clinic letters were used. Extensive ILD was defined by moderate–severe parenchymal lung disease or by an FVC ≤70% if mild parenchymal disease was noted on CT [26]. Clinically important COPD was described by a forced expiratory volume in 1 s (FEV_1_)/FVC of <0.7 with an FEV_1_ <60% predicted. Patients with any of the above were deemed to have CLD. Patients with pre-capillary PH but without evidence of CLD (including those with minor or mild parenchymal lung disease on CT) were classified into the PAH group. Patients’ original haemodynamic and clinical classification was used throughout the study.

### Statistical analysis

Data were analysed using R software (v.4.0.5). Data were presented as mean±sd or median (interquartile range) as appropriate. ANOVA or Kruskal–Wallis tests with Bonferroni *post hoc* correction was used to assess differences in patient characteristics between groups. Correlations were assessed using the Pearson method. Diagnostic thresholds were assessed by receiver operating characteristic (ROC) curve analysis using pROC (v.1.18.5) and ggplot2 (v.3.5.0) packages. Long's test was used to assess for significant difference between ROC curves. Steiger's t-test was used to compare correlation coefficients. A paired t-test was used to compare ECSC and GLI-derived predicted values.

## Results

### Baseline characteristics

**FIGURE 1:**
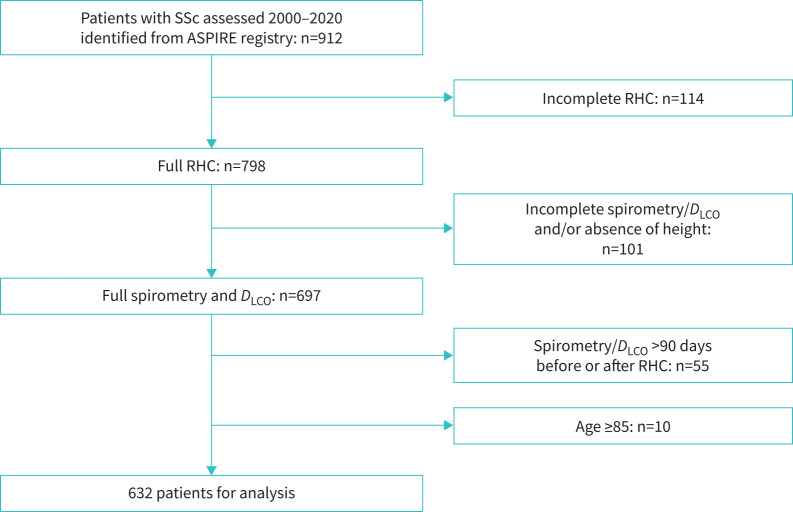
Study flow chart. SSc: systemic sclerosis; RHC: right heart catheterisation; LHD: left heart disease; *D*_LCO_, diffusing capacity for carbon monoxide.

Out of 912 SSc patients assessed during the study period, 798 patients with adequate RHC data were identified (figure 1). Spirometry and gas transfer measures were available for 697 patients. 55 patients had lung function performed >90 days before or after RHC, while in 10 patients ≥85 years old, predictive lung function equations could not be used, leaving a total study cohort of 632 patients.

Baseline characteristics are shown in table 1. 79 patients (13%) had no-PH, 313 (49%) had PAH, 166 (26%) had PH-CLD, 30 (5%) had unclassified-PH and 44 patients (7%) had PH-LHD. The majority of patients (84%) were female with a mean age of 66.1±10 years. Although a higher proportion of patients with PAH and PH-CLD were in World Health Organization (WHO) functional class (FC) III and IV, around half of patients with no-PH or unclassified-PH were also in these higher FC groups. Exercise capacity was worse in patients with pre-capillary PH or PH-LHD than those with no-PH or unclassified-PH. Patients with PH-CLD and PH-LHD had greater spirometric impairment than patients in the other three groups. Whereas values of *K*_CO_% in patients with PAH and PH-CLD were similar (and significantly lower than in patients with no-PH, unclassified-PH and PH-LHD), *D*_LCO_% was lower in PH-CLD than in PAH but similar to PH-LHD. FVC%/*D*_LCO_% was similar in patients with PAH and PH-CLD and higher than in the other three groups. Patients with unclassified-PH had a higher PAWP than patients with pre-capillary PH or no-PH. Compared with ECSC-derived data, GLI-derived % predicted lung volumes were lower (FEV_1_ 81.2±20% *versus* 84.5±20% (p<0.001), FVC 87.1±22% *versus* 94.3±24% (p<0.001)), *D*_LCO_% and *K*_CO_% were higher (45.5±18% *versus* 40.7±15% (p<0.001) and 58.9±20% *versus* 56.8±19% (p<0.001)) and consequently FVC%/*D*_LCO_% was lower (2.16±0.94 *versus* 2.61±1.2 (p<0.001)). Baseline characteristics of subgroups based on the presence or absence of lung disease are summarised in supplementary table S1.

**TABLE 1:** Baseline characteristics

	Whole cohort (n=632)	No-PH (n=79)	PAH (n=313)	PH-CLD (n=166)	Unclassified-PH (n=30)	PH-LHD (n=44)	p-value
**Female, %**	84	90^¶^	89^¶^	71^*#^	77	91	<0.001
**WHO FC I/II/III/IV (%)**	2/16/74/8	8/42/50/0^#¶§^	2/10/80/7^*¶+^	1/8/76/15^*#+^	3/43/53/0^#¶^		<0.001
**WHO FC I/II/III/IV (%)**	2/16/74/8	8/42/50/0^#¶§^	2/10/80/7^*¶+^	1/8/76/15^*#+^	3/43/53/0^#¶^	3/23/73/3*	
**Age, years**	66.14±10	64.27±11	67.14±10	66.41±10	62.29±12	65.05±12	0.036
**SSc form: lSSc/dSSc/not specified/overlap (%)**	75/8/11/5	59/10/17/14^*#¶+^	85/4/8/4^*¶^	66/17/13/4^#^	77/7/13/3b	68/9/18/5	<0.001
**ISWD, m**	160 (80–270)	270 (140–420)^#¶+^	140 (70–270)^*+^	140 (70–250)^*+^	290 (140–480)^¶§^	140 (80–260)^*+^	<0.001
**mPAP, mmHg**	35.8±14	16.77±2^#¶+§^	40.26±13^*+^	38.13±13^*+^	23.9±2^*#¶§^	38.68±14^*+^	<0.001
**PAWP, mmHg**	10.1±3.9	7.7±3^#¶+§^	9.72±3^*+§^	9.3±3^*+§^	12.4±2^*#¶§^	17.93±2^*#¶+^	<0.001
**PVR, WU**	4.5 (2.7–9.3)	1.8 (1.2–2.4)^#¶§^	6.4 (3.6–11.1)^*+§^	5.4 (3.3–9.7)^*+§^	1.7 (1.6–1.9)^#¶§^	3.3 (1.7–6.7)^*#¶+^	<0.001
**CO, L·min^−1^**	4.78±1.6	5.15±2^#+^	4.47±1^*+^	4.71±1^+^	6.83±2^*#¶^	5.33±2^#+^	<0.001
**CI, L·min^−1^·m^−2^**	2.79±0.8	3.06±1^#¶^	2.65±1^*+^	2.76±1^*+^	3.5±1^#¶+^	2.95±1^§^	<0.001
**FEV_1_/FVC**	0.74±0.10	0.76±0.09	0.73±0.08	0.75±0.13	0.76±0.06	0.75±13	0.014
**ECSC % predicted values**							
FEV_1_%	84.5±20	89±20^¶§^	89.91±18^¶§^	73.9±20^*#+^	88.5±20^¶§^	75.42±20^*,#,+^	<0.001
FVC%	94.3±24	97.9±22^¶§^	101.84±21^¶§^	82.5±24^*#+^	96.3±23^¶§^	78.13±25^*#+^	<0.001
FVC%/*D*_LCO_%	2.61±1.15	1.95±0.63^#¶^	2.75±1.04^*+§^	2.96±1.39^*+§^	1.72±0.42^#¶^	2.06±1.01^#¶^	<0.001
*K*_CO_%	56.8±19	68.84±17^#¶^	53.16±17^*+§^	51.95±19^*+§^	75.9±13^#¶^	65.58±21^#¶^	<0.001
*D*_LCO_%	40.7±15	53.4±16^#¶§^	40.29±12^*¶+^	31.57±12^*#+§^	57.7±15^#¶§^	43.34±16^*¶+^	<0.001
**GLI % predicted values**							
FEV_1_%	81.2±20	87.7±22^¶§^	85.16±17^¶§^	71.12±19^*#+^	89.88±24^¶§^	73.27±23^*#+^	<0.001
FVC%	87.1±22	91.84±24^¶§^	92.21±20^¶§^	76.36±21^*#+^	93.6±25^¶§^	78.39±23^*#+^	<0.001
*K*_CO_%	58.9±21	72.83±18^#¶^	54.99±17^*+§^	53±20^*+§^	80±16^#¶^	69.18±24^#¶^	<0.001
*D*_LCO_%	45.5±18	60.9±19^#¶§^	44.85±14^*¶+^	34.75±14^*#+§^	65.98±21^#¶§^	49.08±19^*¶+^	<0.001
FVC%/*D*_LCO_%	2.16±0.94	1.61±0.54^#¶^	2.24±0.84^*¶+§^	2.5±1.16^*#+§^	1.49±0.37^#¶^	1.78±0.76^#¶^	<0.001

Data are presented as median (interquartile range) or mean±sd. CI: cardiac index; CO: cardiac output; *D*_LCO_: diffusion capacity of the lung for carbon monoxide; FEV_1_: forced expiratory volume in 1 s; FVC: forced vital capacity; ISWD: incremental shuttle walk distance; *K*_CO_: carbon monoxide transfer coefficient; mPAP: mean pulmonary arterial pressure; PAWP: pulmonary arterial wedge pressure; PH: pulmonary hypertension; PAH: pulmonary arterial hypertension; PVR: pulmonary vascular resistance; WHO FC: World Health Organization functional class; ECSC: European Coal and Steel Community; GLI: Global Lung function Initiative; lSSc: limited cutaneous systemic sclerosis; dSSc: diffuse systemic sclerosis. p-values <0.05: *: *versus* no-PH; ^#^: *versus* PAH; ^¶^: *versus* PH-CLD, ^+^: *versus* unclassified-PH, ^§^: *versus* PH-LHD.

### Correlations

**FIGURE 2:**
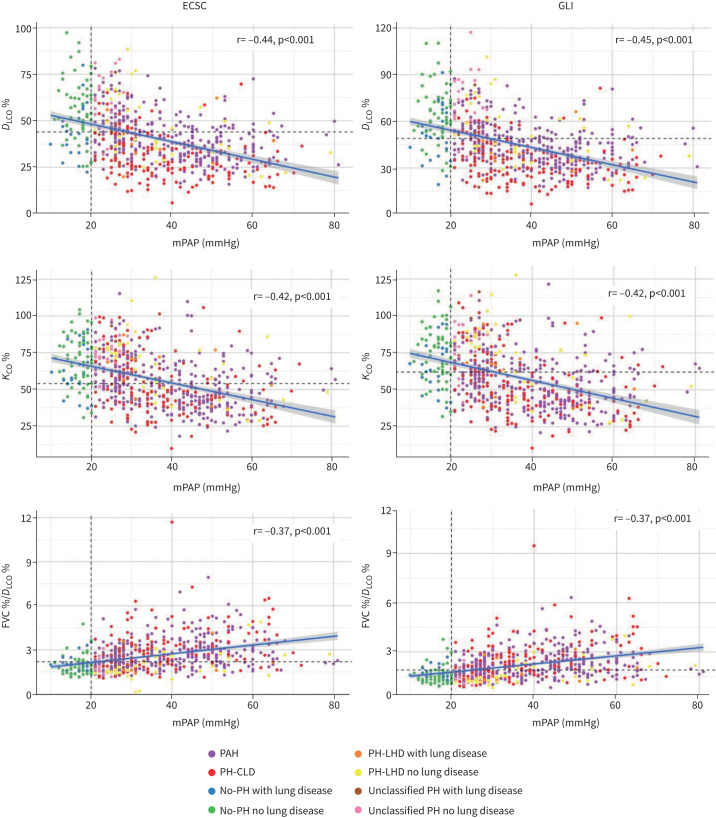
Correlation between mean pulmonary arterial pressure (mPAP) and gas transfer measure. Vertical dashed lines indicate the diagnostic threshold for pulmonary hypertension (PH). Horizontal dashed lines indicate the optimal threshold identified at receiver operating characteristic curve analysis. ECSC: European Coal and Steel Community; GLI: Global Lung Initiative; *D*_LCO_: diffusion capacity of the lung for carbon monoxide; *K*_CO_: carbon monoxide transfer coefficient; FVC: forced vital capacity; PAH: pulmonary arterial hypertension; CLD: chronic lung disease; LHD: left heart disease.

Correlations between the three measures of gas transfer using ECSC and GLI reference values are shown in table 2 and figure 2. Using GLI reference values, *D*_LCO_% had the numerically strongest and FVC%/*D*_LCO_% had the weakest correlation with mPAP in the whole cohort and in those patients with or without significant lung disease (supplementary figure S1a, b). A very similar pattern was observed when using ECSC data.

**TABLE 2:** Correlations of mean pulmonary arterial pressure and % predicted gas transfer measurements

	Overall cohort		No lung disease		Lung disease	
	r	p-value	r	p-value	r	p-value
**ECSC**						
*D*_LCO_%	−0.44	<0.001	−0.51*	<0.001	−0.39	<0.001
*K*_CO_%	−0.42	<0.001	−0.44	<0.001	−0.39	<0.001
FVC%/*D*_LCO_%	0.37	<0.001	0.37	<0.001	0.38	<0.001
**GLI**						
*D*_LCO_%	−0.45	<0.001	−0.51*	<0.001	−0.41	<0.001
*K*_CO_%	−0.42	<0.001	−0.44	<0.001	−0.39	<0.001
FVC%/*D*_LCO_%	0.37	<0.001	0.38	<0.001	0.39	<0.001

*D*_LCO_: diffusion capacity of the lung for carbon monoxide; *K*_CO_: carbon monoxide transfer coefficient; FVC: forced vital capacity; ECSC: European Coal and Steel Community; GLI: Global Lung Initiative. *: significant differences (p<0.05) using Steiger's t-test: *D*_LCO_% *versus* K_CO_%.

### Diagnostic utility

**FIGURE 3:**
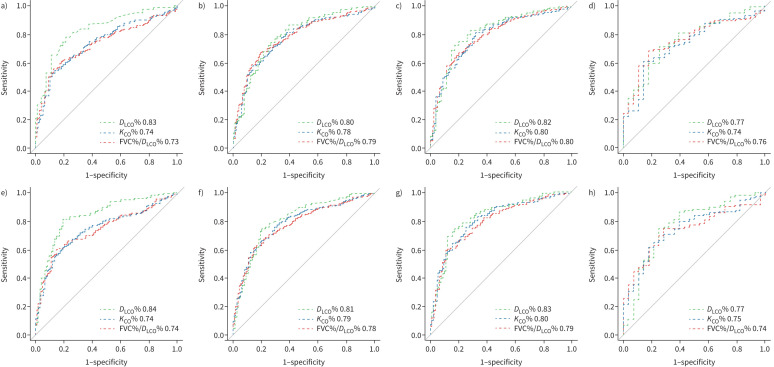
Receiver operator characteristic curve analysis. a, e) Pulmonary arterial hypertension (PAH), pulmonary hypertension (PH)-chronic lung disease (CLD) and PH-left heart disease *versus* no-PH/unclassified-PH. b, f) Pre-capillary PH *versus* no-PH/unclassified-PH. c, g) PAH *versus* no-PH/unclassified-PH (no lung disease). d, h) PH-CLD *versus* no-PH/unclassified-PH (lung disease). ECSC: European Coal and Steel Community; GLI: Global Lung Initiative; *D*_LCO_: diffusion capacity of the lung for carbon monoxide; *K*_CO_: carbon monoxide transfer coefficient; FVC: forced vital capacity.

ROC curve analysis is summarised in table 3 and figure 3. As our previous work demonstrated very similar survival of patients with no-PH and unclassified-PH, for the purposes of ROC analyses the two groups were amalgamated [16]. Almost identical results were achieved if the unclassified-PH patients were excluded from analysis (data not shown). For the overall study cohort, the optimal threshold and area under the curve (AUC) using ECSC data for the prediction of PH for *D*_LCO_% was 44% (AUC 0.83), for *K*_CO_% was 54% (AUC 0.74) and for FVC%/*D*_LCO_% was 2.19 (AUC 0.73: AUC for *D*_LCO_% *versus K*_CO_% or FVC%/*D*_LCO_%, both p<0.001). Using GLI data, the optimal threshold and AUC for the prediction of PH for *D*_LCO_% was 49% (AUC 0.84), for *K*_CO_% was 60% (AUC 0.74) and for FVC%/*D*_LCO_% was 1.91 (AUC 0.74: AUC for *D*_LCO_% *versus K*_CO_% or FVC%/*D*_LCO_% both p<0.001). AUCs for *D*_LCO_% remained numerically superior in analysis of subgroups of patients with pre-capillary haemodynamics and the presence or absence of lung disease. ROC analysis using the previous haemodynamic definition of pre-capillary PH (mPAP ≥25 mmHg and PVR >3 WU) produced almost identical results (data not shown). Only six patients with pre-capillary PH had a *D*_LCO_% (GLI) >80% (sensitivity 99%, specificity 17%), whereas no patients with pre-capillary PH had a *D*_LCO_% (ECSC) of >80% (sensitivity 100%, specificity 6%). Conversely, only three patients with unclassified-PH in the absence of lung disease had a *D*_LCO_% lower than the optimal threshold (47% ECSC and 53% GLI).

**TABLE 3:** Diagnostic utility of % predicted gas transfer measurements

	*D*_LCO_%				*K*_CO_%				FVC%/*D*_LCO_%				p-value
	AUC	Threshold (%)	Sens	Spec	AUC	Threshold (%)	Sens	Spec	AUC	Threshold	Sens	Spec	
**ECSC**													
Pre-capillary PH and PH-LHD *versus* no/unclassified-PH	0.83	44	0.81	0.75	0.74	54	0.55	0.86	0.73	2.19	0.61	0.82	<0.001* <0.001^#^ 0.62^¶^
Pre-capillary PH *versus* no/unclassified-PH	0.80	46	0.77	0.72	0.78	54	0.58	0.86	0.79	2.15	0.66	0.81	0.25* 0.54^#^ 0.54^¶^
PAH *versus* no/unclassified-PH (no lung disease)	0.82	47	0.75	0.80	0.80	68	0.82	0.68	0.80	2.15	0.67	0.80	0.18* 0.26^#^ 0.97^¶^
PH-CLD *versus* no/unclassified-PH (lung disease)	0.77	37	0.72	0.75	0.74	54	0.61	0.86	0.76	2.14	0.69	0.82	0.38* 0.86^#^ 0.44^¶^
**GLI**													
Pre-capillary PH and PH-LHD *versus* no/unclassified-PH	0.84	49	0.81	0.81	0.74	60	0.62	0.80	0.74	1.91	0.60	0.85	<0.001* <0.001^#^ 0.67^¶^
Pre-capillary PH *versus* no/unclassified-PH	0.81	50	0.75	0.80	0.79	67	0.77	0.69	0.78	1.90	0.59	0.85	0.25* 0.54^#^ 0.54^¶^
PAH *versus* no/unclassified-PH (no lung disease)	0.83	53	0.75	0.83	0.80	71	0.83	0.67	0.79	1.90	0.59	0.89	0.18* 0.26^#^ 0.97^¶^
PH-CLD *versus* no/unclassified-PH (lung disease)	0.77	42	0.75	0.75	0.75	58	0.65	0.79	0.74	1.72	0.75	0.71	0.38* 0.86^#^ 0.44^¶^

*D*_LCO_: diffusion capacity of the lung for carbon monoxide; FVC: forced vital capacity; *K*_CO_: carbon monoxide transfer coefficient; PH: pulmonary hypertension; PAH: pulmonary arterial hypertension; AUC: area under the curve; ECSC: European Coal and Steel Community; GLI: Global Lung Initiative; Sens: sensitivity; Spec: specificity. p-values: *: *D*_LCO_% *versus K*_CO_%. ^#^: *D*_LCO_% *versus* FVC%/*D*_LCO_%. ^¶^: *K*_CO_% *versus* FVC%/*D*_LCO_%.

## Discussion

By interrogating a large registry of SSc patients referred with suspected pre-capillary PH we have been able to compare the correlation with pulmonary arterial pressure and diagnostic utility of three measures of gas transfer (*D*_LCO_%, *K*_CO_% and FVC%/*D*_LCO_%). We have also compared the effect of changing from the ECSC to GLI predictive equations.

We observed that, in the overall study cohort and in the subgroups with or without significant lung disease, correlations with mPAP were non-inferior (and were indeed numerically strongest) for *D*_LCO_% and numerically weakest for FVC%/*D*_LCO_%. We observed correlations between *D*_LCO_% and mPAP of r= −0.44 (ECSC) or −0.45 (GLI) in the overall cohort and −0.51 (both equations) in patients without significant lung disease, which are stronger than the r=0.30 reported by Mukerjee *et al.* [27] in 85 SSc patients without significant fibrosis. Sivova *et al.* [28] studied 63 SSc patients and observed correlations between systolic pulmonary arterial pressure (derived from echocardiography) and *D*_LCO_% and FVC%/*D*_LCO_% of r= −0.67 and r=0.66, respectively. It must be noted that only a minority of their study (n=18) had PH.

We also observed that, within the overall study cohort, the AUC for *D*_LCO_% was significantly higher than for both *K*_CO_% and FVC%/*D*_LCO_%. In addition, AUC for *D*_LCO_% was numerically stronger than for *K*_CO_% and FVC/*D*_LCO_% in the subgroup of patients with lung disease. A small number of previous studies have reported a range of AUCs for the presence of PH in patients with SSc (ranging from 0.56 to 0.93 for *D*_LCO_% and from 0.54 to 0.93 for FVC%/*D*_LCO_%) [6, 12, 28, 29]. The wide range of AUCs likely represents differences in patient characteristics between the studies as well as changes in PH haemodynamic definitions. Of note, Soumagne *et al.* [12] studied 572 patients with systemic sclerosis (of whom 336 had no ILD, 226 had ILD and 58 were diagnosed with PH as defined by a mPAP ≥25 mmHg). Similarly to our study, they did not observe any superiority of the diagnostic utility of FVC/*D*_LCO_% over *D*_LCO_% alone in patients with or without ILD (no ILD: AUC for *D*_LCO_% was 0.91 *versus* 0.87 for FVC%/*D*_LCO_%, ILD: AUC for *D*_LCO_% was 0.76 *versus* 0.73 for FVC%/*D*_LCO_%).

Although *K*_CO_ is often described as *D*_LCO_ “adjusted for” *V*_A_, *D*_LCO_ is actually the pressure and unit-adjusted product of *K*_CO_ and *V*_A_. It is therefore apparent that conditions differentially affecting *K*_CO_ and *V*_A_ may result in the same *D*_LCO_ [8]. SSc may be associated with a reduced *K*_CO_ as a result of microcirculatory damage with reduced capillary blood volume (*V*_c_) or increased thickness of the alveolar–capillary membrane with reduced membrane diffusing capacity (*D*_m_) or a combination of the two. ILD may result in reduced *V*_A_, also resulting in lower *D*_LCO_. It has been hypothesised that the ratio of FVC% and *D*_LCO_% may be a more powerful marker of pulmonary vascular disease, especially in patients with ILD, as the *D*_LCO_ will fall disproportionately compared with any fall in FVC resulting in an increased ratio [9]. The reason for the lack of a demonstrable superiority *in vivo* of FVC%/*D*_LCO_% over *D*_LCO_%, in terms of both correlation with mPAP and of diagnostic utility, is not clear. One could postulate that it relates to the fact that an ILD-related reduction in FVC will also reduce *V*_A_, resulting in a correlation between the numerator and denominator.

In 1993 the ECSC reference values for pulmonary function tests were recommended for use by a ERS clinical statement [13, 21]. These reference values were derived purely from European males working in coal mines and steel works and it is important to note that approximately 80% of patients with SSc are female. The GLI subsequently published new reference values, based on a sex-balanced and more geographically diverse patient cohort, for spirometry and *D*_LCO_ in 2012 and 2017, respectively [14, 24]. Although GLI data initially included ethnicity-specific equations, the subsequent GLI Global equations used in our study are race-neutral [30].

In addition to the study of Soumagne *et al.* [12] discussed above, Mangseth *et al.* [31] subsequently studied 577 non-SSc patients (pulmonary fibrosis, haematology disorders, lung transplant recipients and healthy controls) who had taken part in clinical studies. Both studies observed significantly lower FEV_1_% and FVC%, higher *D*_LCO_% and hence lower FVC%/*D*_LCO_% predicted values using GLI equations when compared with ECSC. We replicated these findings in an independent large cohort of SSc patients. These observations are clinically important in terms of longitudinal follow-up of patients with historical ECSC data, but also in identifying patients who require further investigations. For example, FVC%/*D*_LCO_% is one of six parameters in step 1 of the DETECT algorithm, while *D*_LCO_ <70% predicted and FVC%/*D*_LCO_% ≥1.8 are criteria for further investigation in the ASIG algorithm [6, 7, 32]. Both these algorithms were derived before the development of the GLI and so the use of GLI-derived values for a patient raises the risk of a patient with PH not proceeding for further investigation due to a falsely reassuring reading.

### Limitations

Lung function data was not always performed at the RHC visit. However, we excluded patients with more than 90 days between RHC and lung function testing. Although *D*_LCO_ measurements were not corrected for haemoglobin levels, this reflects widespread clinical practice when recent haemoglobin levels are unavailable at the time of lung function testing. Anaemia may reduce *D*_LCO_ and affect correlation and AUC measurements but the impact on either ECSC or GLI predicted values would be similar. Although there were a number of patients without PH at RHC, this study cohort consists of patients in whom PH has been suspected which affects its generalisability of its findings to unselected SSc populations. Nevertheless, our observations are consistent with previous, less selected, studies.

### Conclusion

*D*_LCO_% is at least non-inferior to *K*_CO_% or FVC%/*D*_LCO_% in terms of correlations with mPAP and diagnostic utility in patients with systemic sclerosis and suspected PH. Transitioning to GLI equations leads to lower predicted spirometric volumes, higher *D*_LCO_% and hence lower FVC%/*D*_LCO_%. This should be considered when interpreting changes in values over time and when using screening algorithms.

## Supplementary material

**Please note:** supplementary material is not edited by the Editorial Office, and is uploaded as it has been supplied by the author. Supplementary material 00798-2025-supp-fig-1A Supplementary material 00798-2025-supp-fig-1B Supplementary material 00798-2025-supp-Tab-1
