# Supplementary material for: Diffusing capacity of the lung for carbon monoxide, transfer coefficient of the lung for carbon monoxide and forced vital capacity/diffusing capacity of the lung for carbon monoxide in suspected systemic sclerosis-associated pulmonary hypertension: insights from the ASPIRE registry
Source: ERJ Open Res. 2026 Mar 23;12(2):00798-2025. doi: 10.1183/23120541.00798-2025 (PMC13006901; doi:10.1183/23120541.00798-2025)
Supplement: Supplementary file 2 [file 00798-2025-supp-fig-1B.pdf]

Supplementary figure 1B. Correlation between gas transfer measures and mean pulmonary arterial pressure, split by presence or absence of lung disease (GLI)

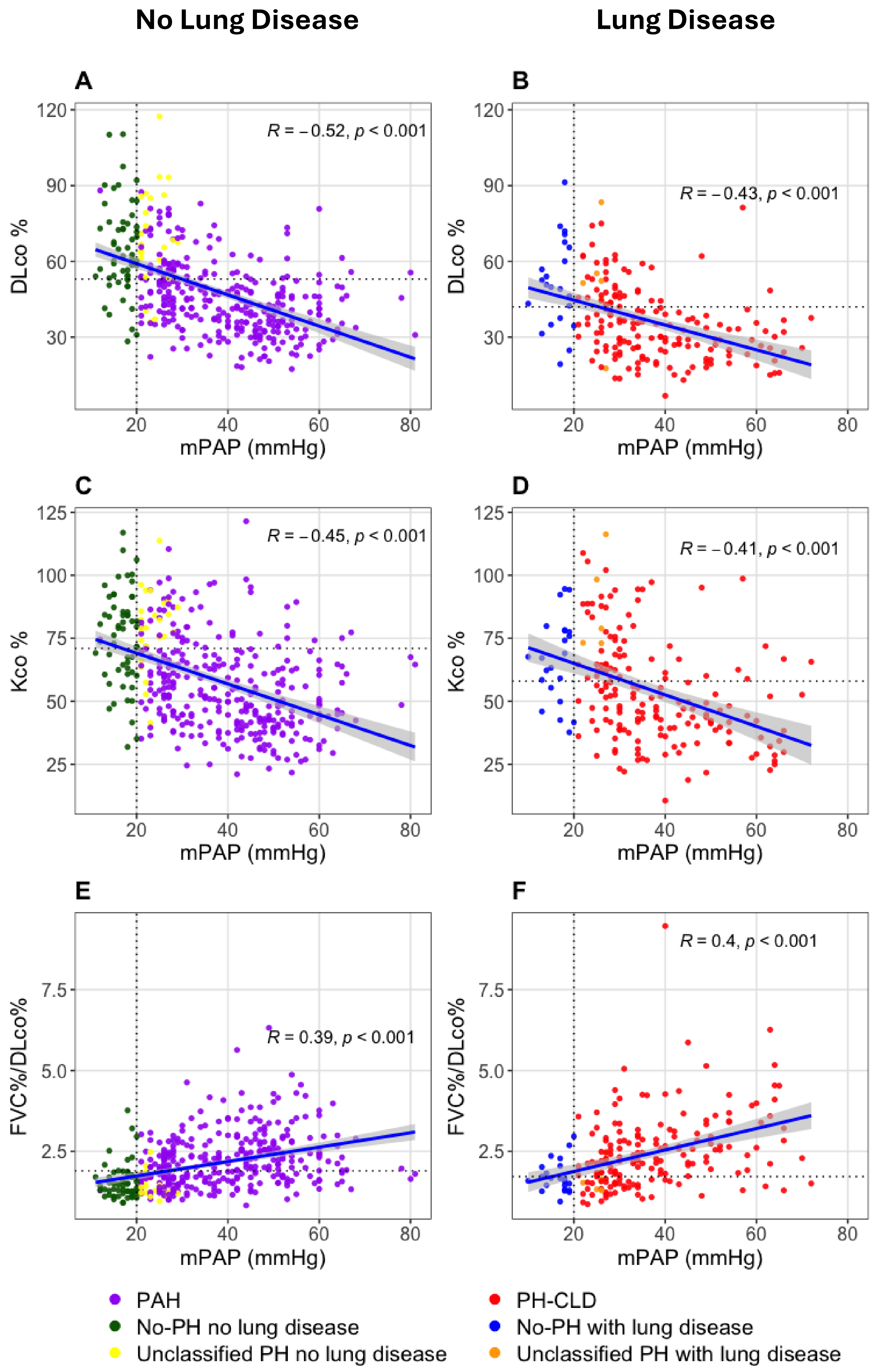

Vertical dashed lines refer to diagnostic threshold for pulmonary hypertension, horizontal dashed lines refer to optimal threshold identified at ROC curve analysis. Abbreviations: DLco, diffusion capacity of the lung for carbon monoxide; FVC, forced vital capacity; Kco, carbon monoxide transfer coefficient; mPAP, mean pulmonary arterial pressure; GLI, Global Lung function Initiative; PAH, pulmonary arterial hypertension; PH-CLD, pulmonary hypertension associated with chronic lung disease.
