# Supplementary material for: Diffusing capacity of the lung for carbon monoxide, transfer coefficient of the lung for carbon monoxide and forced vital capacity/diffusing capacity of the lung for carbon monoxide in suspected systemic sclerosis-associated pulmonary hypertension: insights from the ASPIRE registry
Source: ERJ Open Res. 2026 Mar 23;12(2):00798-2025. doi: 10.1183/23120541.00798-2025 (PMC13006901; doi:10.1183/23120541.00798-2025)
Supplement: Supplementary file 3 [file 00798-2025-supp-Tab-1.pdf]

Supplementary table S1. Baseline characteristics stratified by presence or absence of chronic lung disease

	**No-PH, No CLD (n = 57)**	**No-PH with CLD (n = 22)**	**Unclassified-PH, No CLD (n = 24)**	**Unclassified-PH with CLD (n = 6)**	**PAH (n = 313)**	**PH-CLD (n = 166)**	**PH-LHD, No CLD (n = 34)**	**PH-LHD with CLD(n = 10)**	**p-Value**
Female (%)	91	86	79 [g]	67 [g]	89 [f]	71 [e]	100 [c d]	60	<0.001
WHO FC I/II/III/IV (%)	7/41/52/0 [e f g]	9/45/45/0 [d f g]	4/44/52/0 [e f]	0/33/66/0 [a]	2/10/80/7 [a b c f g]	1/9/75/16 [b c e g]	3/24/60/3 [b e]	0/10/90/0 [a b f]	<0.001
Age (years)	63.5 ± 11	66.13 ± 10	61.12 ± 12	66.97 ± 10	67.1 ± 10	66.41 ± 10	64.16 ± 13	68.04 ± 8	0.053
SSc Form lSSc/dSSc/Not Specified/Overlap (%)	75/8/11/5	59/10/17/14 [e]	85/4/8/4	66/17/13/4	77/7/13/3 [b]	68/9/18/5	71/6/18/6	60/20/20/0	0.694
ISWD (metres)	275 (135, 410) [e f]	265 (180, 435) [e f]	340 (100, 245) [e f]	195 (180, 560)	140 (70, 270) [a b c]	140 (70, 250) [a b c]	140 (80, 250)	220 (40, 270)	<0.001
mPAP (mmHg)	16.9 ± 2 [e f g h]	16.4 ± 3 [e f g h]	23.5 ± 3 [e f g h]	25.3 ± 2 [e g h]	40.3 ± 13 [a b c d]	38.1 ± 12 [a b c]	39.03 ± 15 [a b c]	37.5 ± 13 [a b c]	<0.001
PAWP (mmHg)	7.9 ± 3 [c d e f g h]	7.2 ± 3 [c d e f g h]	12.54 ± 2 [a b e f g h]	12 ± 2 [a b g h]	9.7 ± 3 [a b c g h]	9.3 ± 3 [a b c g h]	17.94 ± 2 [a b c d e f]	17.9 ± 2 [a b c d e f]	<0.001
PVR (WU)	1.8 (1.3, 2.4) [e f g]	1.9 (1.2, 2.4) [e f]	1.7 (1.4, 1.9) [e f]	1.8 (1.8, 1.9) [e f]	6.4 (3.6, 11.1) [a b c d g]	5.4 (3.3, 9.7) [a b c d]	3.5 (1.6, 6.8) [a e f]	2.9 (2, 4.4)	<0.001
CO (L/min)	5.2 ± 2 [c d e]	5 ± 1 [c d]	6.7 ± 1 [a b e f g]	7.23 ± 2 [a b e f]	4.47 ± 1 [a c d g h]	4.7 ± 1 [c d]	5.32 ± 2 [c e]	5.34 ± 2 [e]	<0.001
CI (L/min/m^2)	3.05 ± 1 [e]	3.07 ± 1	3.53 ± 1 [e f]	3.4 ± 1	2.65 ± 1 [a c]	2.76 ± 1 [c]	2.95 ± 1	2.94 ± 1	<0.001
FEV_1/FVC	0.75 ± 0.9 [f]	0.78 ± 0.9	0.76 ± 0.6	0.77 ± 0.6	0.73 ± 0.8	0.75 ± 0.13 [a]	0.75 ± 0.10	0.74 ± 0.20	0.293
**ECSC percent predicted values**									

FEV$_1$%	91.9 ± 20 [f g h]	81.63 ± 20	91.7 ± 16 [f]	75.5 ± 30	89.8 ± 18 [g f]	73.9 ± 20 [a c e]	76.56 ± 20 [a e]	71.54 ± 22 [a]	<0.001
FVC%	102 ± 21 [f g]	87.1 ± 23 [e]	100.5 ± 18 [f g]	79.14 ± 35	101.9 ± 21 [b f g]	82.5 ± 24 [a c e]	77.56 ± 27 [a c e]	80.07 ± 21	<0.001
K_{CO}%	70.6 ± 17 [e f]	64.2 ± 17 [c f]	75.14 ± 14 [e f]	78.9 ± 10 [e f]	53.2 ± 17 [a c d g]	52 ± 19 [a b c d g]	66.11 ± 22 [e f]	63.81 ± 15	<0.001
DL_{CO}%	57.8 ± 16 [b e f h]	44.6 ± 15 [a c f g]	60.4 ± 14 [b e f g h]	46.8 ± 16	40.4 ± 12 [a c f]	31.6 ± 12 [a b c e g]	44.77 ± 17 [b c f]	38.45 ± 13 [a c]	<0.001
FVC%/DL_{CO}%	1.91 ± 0.64 [e f]	2.06 ± 0.61 [f]	1.74 ± 0.46 [e f]	1.67 ± 0.20	2.75 ± 1.04 [a c g]	2.96 ± 1.39 [a b c g]	1.99 ± 1.02 [e f]	2.28 ± 0.99	<0.001
**GLI percent predicted values**									
FEV$_1$%	89.78 ± 21 [f g h]	82.23 ± 21	93.04 ± 23 [f g h]	77.26 ± 27	85.2 ± 17 [f]	71.12 ± 19 [a c e]	74.92 ± 24 [a c]	67.69 ± 20 [a c]	<0.001
FVC%	94.84 ± 22 [f g]	84.09 ± 27	97.42 ± 23 [f]	78.31 ± 29	92.3 ± 20 [f g]	86.36 ± 21 [a c e]	80.06 ± 25 [a e]	72.71 ± 16	<0.001
Kco%	75.24 ± 18 [e f]	65.58 ± 17 [e f]	79.08 ± 15.37 [e f]	83.65 ± 20 [e f]	55.1 ± 17 [a c d g]	53.01 ± 20.25 [a b c d g]	70.87 ± 24.78 [e f]	63.43 ± 20.79	<0.001
DLco%	64.6 ± 18 [b e f g h]	51.29 ± 18 [a c f]	69.76 ± 19 [b e f g h]	50.8 ± 21	45 ± 14 [a c f]	34.8 ± 14 [a b c e g]	51.33 ± 20 [a c f]	41.44 ± 13 [a c]	<0.001
FVC%/DLco%	1.56 ± 0.55 [e f]	1.72 ± 0.50 [f]	1.45 ± 0.35 [e f]	1.64 ± 0.42	2.24 ± 0.84 [a c f]	2.5 ± 1.16 [a b c e g]	1.75 ± 0.78 [f]	1.91 ± 0.72	<0.001

Abbreviations: CI, cardiac index, CO cardiac output; DLco, diffusion capacity of the lung for carbon monoxide; FEV$_1$, forced expiratory volume in 1 second; FVC, forced vital capacity, ISWD, incremental shuttle walk distance; K_{CO}, carbon monoxide transfer coefficient; DL_{CO}, diffusion capacity of the lung for carbon monoxide; mPAP, mean pulmonary arterial pressure; PAWP, pulmonary arterial wedge pressure; PH, pulmonary hypertension,; PAH, pulmonary arterial hypertension; PVR, pulmonary vascular resistance; WHO FC, World Health Organisation functional class; ECSC, European Coal and Steel Community; GLI, Global Lung function Initiative.

p-values <0.05: a = versus No PH; b = versus No PH with CLD c = versus Unclassified-PH, d = versus unclassified-PH with CLD, e = versus PAH, f = versus PH-CLD, g = PH-LHD without CLD, h = PH-LHD with CLD.
